# Monkeypox: Potentially another pandemic or a mere hoax?

**DOI:** 10.1016/j.amsu.2022.104364

**Published:** 2022-08-18

**Authors:** Sajeel Saeed, Haroon Shabbir, Jawad Basit, Mohammad Ebad ur Rehman

**Affiliations:** Department of Medicine, Rawalpindi Medical University, Pakistan

## Abstract

Monkeypox is a vesicular, pustular illness caused by an orthopox virus. It has remained endemic to the Central and West African regions for the past four decades, with only a few outbreaks outside these regions. It is mainly transmitted by direct skin to skin with an infected individual who has developed pustules. Recently there have been major outbreaks in about fourteen countries of the world, and over nine hundred people have been infected. This short summary gives a review about the epidemiology, history, virulence, symptoms and prevention of Monkeypox, while addressing a major question that the general public has right now: “Will Monkeypox turn into the next global pandemic?”.

As the globe heals from the century's epidemic, a new virus, Monkeypox, seems to be surfacing. Until recently, more than 900 suspected and confirmed instances of monkeypox have been documented; a normally mild sickness that spreads via intimate contact and causes flu-like symptoms and a characteristic rash [1]. The majority of cases have occurred in Europe rather than in the endemic areas of Central and West Africa [2]. In the present epidemic, no fatalities have been recorded. The public is understandably worried about the emergence of yet new ailment that they may have never heard of before. “Will this be as horrible as Covid?” is a question that many individuals may have.

Monkeypox is a zoonotic orthopox virus that causes a vesicular, pustular illness similar to smallpox [3]. The incubation period is usually seven to 14 days but can range from five to 21 days. The illness typically lasts for two to four weeks. The salient difference between Monkeypox and Smallpox is that Monkeypox presents with lymphadenopathy in the later stages [3]. Monkeypox was first isolated in 1958 in monkeys, but the first human case was confirmed in the Democratic Republic of Congo (DRC) in 1970 [2,4]. Monkeypox has been prevalent in West and Central Africa for more than four decades, with rodents as its primary host. The Central African strain has undergone much more human-to-human transmission than the West African strain. Human cases have been reported in places other than Africa, including a significant epidemic in the United States in 2003 that infected fifty-three persons [3,4].

In comparison to COVID-19, Monkeypox does not seem to be as easily transmissible. Transmission can occur via saliva, respiratory droplets, or by close skin to skin contact with someone who has the lesions caused by Monkeypox. In contrast, COVID-19 could be spread from person to person even if they were standing as far as 5 ft apart [5]. Apart from the lower rate of transmission of Monkeypox as compared to COVID, Monkeypox also has a lower Case Fatality Ratio (CFR). The smallpox vaccine seems to be effective in preventing Monkeypox, and mortality rates have been higher (up to 11%) in people unvaccinated for smallpox. However, as smallpox has been eradicated in most parts of the world, its vaccination has been discontinued. This has led to an increased predisposition to Monkeypox in people who have not received the smallpox vaccine. Mortality in COVID is attributed to development of respiratory distress, pulmonary edema or even septic shock in the later stages of disease, whereas the characteristic ailments in Monkeypox infection include an initial febrile prodrome and rash, later accompanied by secondary bacterial infection and painful, tender lymphadenopathy [2]. The genomic material in the two viruses is different: coronaviruses have RNA and poxviruses have DNA [6]. This is a key distinction, because RNA viruses tend to mutate more easily when they reproduce than DNA viruses, which have more stable genomes. This feature has allowed Covid to continue to spread in multiple waves across the world with each new variant. There are no currently approved treatment options for Monkeypox, hence prevention by “Ring Vaccination” using the smallpox vaccine is advised, which involves vaccinating close contacts of individuals who get infected [7].

In terms of the present epidemic, the first case of monkey pox was discovered on May 7, 2022 in the United Kingdom. More than 900 instances have been verified by the June 4, 2022, with another 71 cases suspected [1]. Monkeypox had involved around 43 nations all around the world. The major countries which are infected include United Kingdom, Spain, Portugal, Canada, Germany, France, Netherlands, United States of America, and Italy [1]. Most of the cases are found in United Kingdom. Owing to this, the National Health Services (NHS) of England has also devised some preventive measures to stop the spread of Monkeypox [8]. According to the NHS, persons who have had close contact with infected individuals, who have recently been to Central or West Africa, and who have had intercourse with males are more likely to get this disease [8].

Multiple causes have brought monkeypox to the point of becoming a large viral epidemic. Each year, hundreds of cases of monkeypox are reported in Central Africa, and rodents are the primary vectors for their transmission. The travel history of the first monkeypox case detected outside of Europe was traced to Nigeria [4]. Moreover, while these cases are mostly identified in people aged 20 to 50, it may be because smallpox immunization was discontinued in the 1980s, when the incidence of monkeypox in Central Africa was very low [7]. Moreover, while the majority of monkeypox cases were detected in men, the likely cause identified in the diagnosed cases was sexual activity with male partners [8]. Unknown is the likely cause of this sexual transmission. The United Kingdom's national health services (NHS) also encouraged all patients to coordinate with sexual health professionals if they have symptoms [8].

Though lower transmission rate and lower case fatality ratio suggest that if the appropriate preventive measures are adopted in the early stages of this disease's spread, there is little likelihood that this will develop into the next global pandemic, the rapid increase in the number of Monkeypox cases over the past month has caused alarm around the globe. Although, in comparison to Corona Virus (COVID-19), the number of cases detected during the first month is lower, some measures, such as practising appropriate hygiene, must be taken. The antiviral tecovirimat for treating seriously ill monkeypox cases, as well as the third-generation smallpox vaccine for use as prophylaxis in all close and high-risk case contacts, as well as specific guidelines for their use, must be made universally available at an affordable cost as soon as possible [9].

## Please state any conflicts of interest

All authors declared no conflict of interest.

## Please state any sources of funding for your research

No funding required for the study.

## Ethical approval

Not applicable.

## Consent

Not required.

## Author contribution

Sajeel Saeed: Study conception, write-up, critical review and approval of the final version. Haroon Shabbir: Study conception, write-up, critical review and approval of the final version. Jawad Basit: Study conception, write-up and approval of the final version. Mohammad Ebad ur Rehman: Study conception, critical review and approval of the final version.

## Registration of research studies

1. Name of the registry: Not applicable.

2. Unique Identifying number or registration ID: Not applicable.

3. Hyperlink to your specific registration (must be publicly accessible and will be checked): Not applicable.

## Guarantor

Sajeel Saeed: Department of Medicine, Rawalpindi Medical University, Block E Satellite Town, Rawalpindi, Pakistan.
